# Quantitative Analysis of Internet of Things Technology on the National Economic Accounting: A Prediction Model Based on the *FCM-BP* Neural Network Algorithm

**DOI:** 10.1155/2022/5335310

**Published:** 2022-05-05

**Authors:** Jian-Tao Song

**Affiliations:** School of Finance and Accounting, Yellow River Conservancy Technical Institute, Kaifeng 475004, China

## Abstract

The development of Internet of Things *(IoT*) technology is of great significance for modern financial settlements based on information technology. The *IoT* technology, which can provide a convenient approach for accounting, has the advantages of intelligent processing, reliable transmission, and comprehensive perception. For national economic accounting, a new integrated system for monitoring the environment is developed and designed by using embedded development technology and sensor technology. The system uses a wireless sensor network environment monitoring system *IoT* platform with embedded internal processors. Analyze and design the system as a whole, including the construction of the basic platform of the system, the design of the internal plates and circuits of the system, the monitoring design of the input node, and the monthly design of the output interface calculation. Finally, a physical model is built, and data measurement and analysis are carried out under different conditions, and the evaluation and advantage analysis of the system's operating status are given. The system can carry out all-round, multilevel and three-dimensional real-time monitoring of the construction site environment, including dust, *PM2.5*, temperature, humidity, wind speed, carbon dioxide, and other indicators in the construction site environment. In addition, the system can upload various monitoring data to the detection system through the internal network. The system has the functions of monitoring, alarming, recording, querying, and counting of the target monitoring station and can also be linked with the environmental control device. The construction site staff can conduct real-time supervision through the mobile terminal and computer terminal management platform. In addition, it can also meet the role of real-time remote monitoring and online guidance and regulation. It has reference value for the safety and management of the actual operation process of the project.

## 1. Introduction

The Internet of Things has been applied to industries, medical care, agriculture, logistics, transportation, power grids, environmental protection, fire protection, furniture, and other aspects that are closely related to life. It provides great help in economic development and also provides a reference basis for the writing of this article. The Internet of Things technology has already affected the digital strategy of the central bank in many aspects [1]. The financial management of enterprises directly related to numbers is the first to be affected [2]. With the progress and development of Internet of Things technology, the organization and operation mode of enterprises will also be overturned. Blockchain technology will become an important part of transactions between businesses within the next five years, according to multiple surveys by accounting consulting firms. The *IoT* technology is also affecting accounting and related industries, and just like other existing payment methods. Financial managers need to integrate payment methods into their public affairs management systems and processes, which makes the management system of public goods include a lot of actual economic accounting content [3]. Certified public accountants can even view public business and financial data in real time through public keys, which will improve the public's trust in their corporate financial statements. In particular, it inhibits financial fraud of citizens. Internet of Things technology will push financial management professionals to expand their skills. In addition, the Internet of things is an accounting number processing technology, which can be used to create accounting information management systems, and can also be applied to create multiple immutable audit tracking systems [4]. As the Internet of things technology matures, external auditing can be overturned. Neural network intelligent contract can be applied to automatic payment between banks and enterprises as well as between enterprises and establish bank loan or accounts receivable management system without trust and supervision. Nowadays, the Internet is being used by more and more people. In such an environment, people's demand is also changing constantly. For some production and sales enterprises, customer demand changes greatly, resulting in changes in the production mode of enterprises. In the age of the Internet of Things, digital is the foundation, being used to connect businesses and banks [5–8]. The prevalence of Internet of Things technology has brought about changes in many aspects of national economic accounting, and great changes have taken place in the way of accounting and recording of flows and stocks.

Public assets are the material basis and basic condition for the sustainable development of national economy and also the concentrated embodiment of the level of national productivity. The application of the Internet of Things technology can greatly promote the improvement of the national economic level and play a role in the development of national economic accounting to a large extent, especially under the conditions of a market economy. National asset accounting has always been an important content in macroeconomic research and a prerequisite for macroeconomic analysis such as total factor productivity measurement [9]. Accounts are the basic tool for checking and calculating the economy of the people of the country. The operation process of the national economy can be described by setting different accounts. The classification of the national economy includes agriculture, forestry, animal husbandry, fishery, etc. Accordingly, the frame system of whole national economic accounting and account system keep consistent roughly. Due to the need to cover the whole process of macroeconomic operation, the system of national economic accounting has the characteristics of extensive and complex contents. SNAs are intelligently identified and tracked. The limits on the scope of production units are affected by many factors. This complicates national accounts. Several contents are maintaining stable [10]. With accounting methods getting more mature, accounting system is continuously improved. However, there are more content that has not been unanimously recognized, or accounting methods are in the process of exploration. Accordingly, whole national economic accounting system is divided into two parts, i.e., central frame and satellite account[11].

In the global digital wave, the *IoT* plays an important role in promoting the digital transformation of traditional manufacturing industry and winning the dividend of digital economy. At present, the Internet of Things has been involved in the manufacturing of raw materials, equipment, consumer goods, and other industries, achieving broader, higher-level, and deeper development and forming a variety of comprehensive application practices [12]. More and more enterprises start to build and use the Internet of Things platform, which has become the primary development direction of all industries [13, 14]. With the development of China's economy and society, network technology is becoming more and more mature. In the era of artificial intelligence, cloud computing and Big Data are widely used, and various solutions to bank financial settlement provided by Internet of Things technology are also increasing and gradually improved. At the same time, the country has issued a lot of relevant policy documents, laid a foundation for the development of the network, but also brought opportunities [15].

The rest of this manuscript is organized as follows: Section 2 gives the background and discusses some existing work on Internet of things service composition.  Section 3 introduces the proposed multiagent settlement system for Internet of things service combination.  Section 4 describes the prediction results from SCM-BP model.  Section 5 describes recommendations for actual scenarios and performance evaluation. Finally, Section 6 gives the conclusion and the future prospect of national economic settlement. The purpose of national accounts is to measure economic flows and stocks. The economic flow involves a series of problems. National accounts set out a series of accounting concepts for transactions and other flows, products and production units, assets and liabilities. Relevant information is recorded through a system of relevant accounts, which ultimately produces economic data that can be used to monitor economic activity, macroeconomic analysis, and international comparisons. Among them, the emphasis in this article is on macroeconomic regulation, which is less in international comparisons.

## 2. Related Background

**Figure 1:**
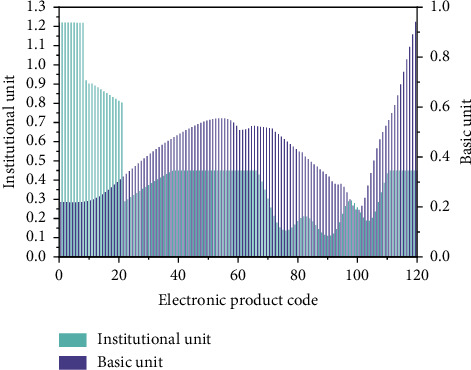
Internet code number in two units.

The concept of the Internet of Things was first proposed in 1999 and applied to asset computing recently. *IoT* technology is a method using information identification to identify and manage the affairs and evolution laws under network conditions [16]. This technology is also called the interconnection between items. The essence of the technology is mainly to effectively integrate *RFID* technology and the Internet and to effectively apply the integrated products. The emergence of the IoT technology can effectively solve the interconnection between different levels of business, which is also quite different from the traditional Internet forms. In the previous literature, the assets settlement process of the national economy is studied by different methods to a great extent.  Figure 1 gives the data of Internet code number in two units, which comes from previous research results [17]. The Internet of Things is a network and data platform that makes comprehensive use of *P2P* (Peer to Peer Lending) network, digital encryption, distributed consensus, smart contract, and other technologies. Its operation and promotion in the economic field need the support of multiple disciplines such as statistics, information management, and agricultural technology [18,19]. To promote the application of Internet of Things technology in the field of digital economic analysis, we must make full use of the existing opportunities and actively respond to the challenges.

**Figure 2:**
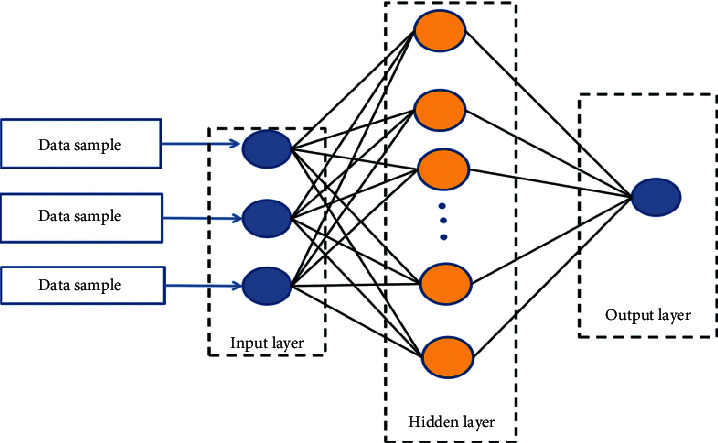
Schematic diagram of the basic framework of neural network.

Neural network and cluster analysis have played an irreplaceable role in the development and application of Internet of Things technology. As can be seen from Figure 2, the *BP* network is a multilayer mapping neural network, which can completely approximate any complex nonlinear relationship. LM back propagation function is a multiple layer feedforward network based on back propagation algorithm, also known as BP Network, which can approximate any continuous function with any accuracy. The *BP* neural network model with a *LM* back propagation function is relatively suitable for this research scene of accounting of the national economy. Besides, the high accuracy can reach a high level after the test data under some typical head being trained. It adopts the error back propagation learning algorithm to adjust the weight and other parameters to achieve the best. It has the advantages of wide adaptability and effectiveness. Due to the uneven values of input nodes, the neural network is ensured to be studied samples and prevented a large amount of information from drowning decimal information. For decades, common prediction models include time series method [20], support vector machine method and neural network method. The basic neural network has high computational accuracy and good global stability, but it takes a long time to learn. It represents a schematic diagram of the basic framework of the neural network. BP neural network is not only short in learning time, but also simple and easy to operate. Previous studies have proved that FCMBP algorithm has less distortion than transitive closure method, and the clustering results of clustering methods are not always the same, and sometimes there are essential differences. In order to improve the accuracy of the forecast, the influential factors in the process of economic forecast were considered comprehensively, and the samples were clustered according to the key features by fuzzy clustering (*FCM*), and the economic forecast model with the same features was established. In the calculation example, the data released by the finance bureau of a province in China from 2008 to 2018 are used for prediction, and compared with the traditional prediction results, respectively, so as to verify the validity of the model proposed in this study. In this paper, evolutionary programming is used to replace the traversal optimization mechanism of FCMBP method to find the fuzzy equivalent matrix with the minimum distortion, and a FCMBP fuzzy clustering method based on evolutionary programming (EP-FCMBP) is proposed. In this method, a group of fuzzy equivalent matrices are generated randomly at first, a new fuzzy equivalent matrix is generated by mutating each equivalent matrix, then the survival of the fittest and population evolution are achieved by selecting the operators, and finally a fuzzy equivalent matrix with minimum distortion is obtained [21].

## 3. Establishment of the Adopted *FCM-BP* Model

### 3.1. Asset Classification Method Based on Fuzzy *C-Means* Clustering

The database and configuration software are adopted to realize the preprocessing and analyzing of the basic data. This system is based on computer, communication equipment, measurement, and control unit as basic tools and provides a basic platform for real-time data acquisition, switch status monitoring, and remote management and control of large public buildings. It can form any complex monitoring system with detection and control equipment. The system mainly adopts a layered distributed computer network structure, which is generally divided into three layers: the station control management layer, the network communication layer, and the field device layer. The fuzzy *C-means* clustering algorithm has been very mature in the field of classification based on features. According to the distance between data points and clustering centers, it obtains the membership degree of each sample point to all class centers. The larger the membership degree is, the closer the distance between data points and clustering centers is.

(1) $$\begin{array}{c} J_{m}=\sum_{i=1}^{N}\sum_{j=1}^{C}u_{ij}^{m}{\left|X_{i}-C_{j}\right|}^{2},\;1\leq m<\infty , \end{array}$$

where the range of *m* is a real number greater than 1; *u*_ij_ represents the membership degree, that is, the membership degree of *X*_i_ in the objective function *j*, *X*_i_ is the *i*th data of *n*-dimensional measurement data, and *C*_i_ is the clustering center of *n* dimension.

Fuzzy classification is found through finite iterations of the above objective function. Meanwhile, membership degree *u*_ij_ and cluster center *C*_i_ are constantly updated through.

(2) $$\begin{array}{c} u_{ij}=\frac{1}{\sum_{k=1}^{C}{\left(d_{ij}/d_{kj}\right)}^{2/\left(m-1\right)}}. \end{array}$$

The fuzzy classification from Internet of Things can effectively solve the interconnection between objects to objects, people to people, and people to objects. The Internet of Things is also quite different from the traditional Internet. For example, the interconnection between people and objects we are talking about mainly refers to the connection between people and objects through the use of some common devices. The interconnection between people mainly means that the process of interconnection between people no longer only depends on the connection method of personal computers.

(3) $$\begin{array}{c} E_{k}=0.5\sum_{i=1}^{q}{\left(Y_{i}^{k}-C_{t}^{k}\right)}^{2}, \end{array}$$

where *E*_*k*_ represents the error between the expected value and the actual value of the neural network.

There are not many related studies on the application of fuzzy *C-means* clustering algorithm to the evaluation of national economic benefits. Therefore, this paper will construct a national economic benefit evaluation system based on fuzzy *C-means* cluster analysis algorithm. Because the quality of national economic benefits itself is ambiguous, it is difficult to directly evaluate the evaluation indicators, but the fuzzy evaluation method can combine qualitative and quantitative. This provides a more objective and feasible method for the evaluation of national economic benefits.

### 3.2. *BP* Neural Network

*BP* neural network is one of the most widely used multilayer feedforward networks. A three-layer *BP* neural network can realize arbitrary precision and approximate arbitrary continuous function. The structure of *BP* neural network mainly includes three layers: input layer, hidden layer, and output layer. Suppose that the neural network input layer is *m*, hidden layer is *l*, and output layer is *n*, and the output of hidden layer is shown as follows:

(4) $$\begin{array}{c} h_{i,l}=f\left(\sum_{p=1}^{m}W_{i,l}^{\left(1\right)}x_{i,p}+b_{i,l}^{\left(1\right)}\right),\;p=1,2,...,m. \end{array}$$

The output layer with input and output is shown as follows:

(5) $$\begin{array}{c} y_{i,l}=f\left(\sum_{q=1}^{l}W_{i,n}^{\left(2\right)}h_{i,q}+b_{i,n}^{\left(2\right)}\right),\;q=1,2,...,l. \end{array}$$

In the neural network, the hidden layer is transmitted to the output layer. If there is no difference between the actual value and the expected output value, the errors of the two are transmitted back to the input layer, and the weights and thresholds of each neuron connection are modified layer.

The application of the *IoT* is based on optical fiber, to form a network of multiple computers to realize the communication and sharing of information. However, the network environment established by the Internet is actually virtual. The emergence of *IoT* technology is to remedy this problem, thereby avoiding the occurrence of other situations such as network interruption. Based on this, we can find that the application of Internet of things engineering has high requirements for relevant equipment and devices. It needs a certain effectiveness and stability to improve the efficiency of fiscal and tax statistics.

In this paper, fuzzy clustering is used to divide the samples, and *BP* neural network is combined to build a prediction model. When measuring the performance of model prediction, the following error indicators are usually adopted:

(1) Mean absolute error rate (MAPE): MAPE not only considers the error between the predicted value and the real value but also shows the ratio of the error to the real value.
  (6) $$\begin{array}{c} \text{MAE}=\frac{1}{n}\sum_{i=1}^{n}\left|x_{i}-y_{i}\right|. \end{array}$$
(2) Mean absolute error (MAE): MAE is a basic index to investigate the error.
  (7) $$\begin{array}{c} \text{MSE}=\frac{1}{n}\sum_{i=1}^{n}{\left(x_{i}-y_{i}\right)}^{2}. \end{array}$$
(3) Mean square root error (RMSE): RMSE is sensitive to outliers.
  (8) $$\begin{array}{c} \text{RMSE}=\sqrt{\frac{1}{n}\overset{n}{\underset{i=1}{\sum }}{\left(x_{i}-y_{i}\right)}^{2}}. \end{array}$$
(4) Mean variance (MSE). MSE will mainly evaluate the stability of the model through the square amplification error with large deviation
  (9) $$\begin{array}{c} \text{MAPE}=\frac{100\text{\%}}{n}\sum_{i=1}^{n}\left|\frac{x_{i}-y_{i}}{y_{i}}\right|, \end{array}$$
where *y*_i_ is the actual value; *x*_i_ is the predicted value; and *n* is the number of data.

Through the analysis, the factors that have a greater impact on the national economy are obtained. In recent years, information such as fixed investment, total population, and number of employees have become important factors affecting the national economy. This paper adopts the method of sensitivity analysis to analyze the absolute sensitivity of each influencing factor to each factor, that is, the derivative of each factor to each parameter. Therefore, taxes and total population can be used as inputs to the *BP* neural network model.

### 3.3. Prediction Method Based on *FCM-BP* Neural Network

**Figure 3:**
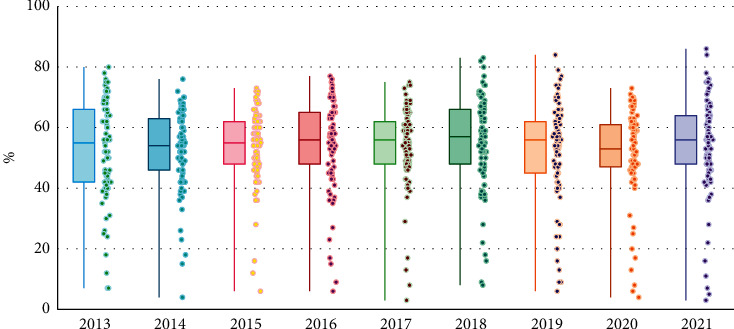
The development process of national economic accounts.

The fuzzy clustering is used to divide the samples, and *BP* neural network is combined to build a prediction model.  Figure 3 shows the development process of national economic accounting. The detailed steps of the *FCM-BP* neural network prediction model are as follows:

(1) Considering the factors of sample difference, the time and weather characteristics are classified and analyzed by using the c-mean of mold paste.
(2) Normalized sample data and the input value of neural network run smoothly in the interval of (0,1).
(3) Adjust the parameters of the neural network and take the normalized sample data and the specific data corresponding to each sample as input; adjust training parameters, including learning rate, training times, and minimum error, etc.
(4) Compare the predicted value of output results with the real value and find out the data with large error for analysis.
  (10) $$\begin{array}{c} s_{k}=\sum_{j=1}^{n}w_{kj}b_{j}-\theta_{k}, \end{array}$$
where *w*_*kj*_ is the connection weight between the *j*th neuron in the hidden layer.

There is no need to analyze the hidden relationship in the original data too much in the early stage, but the hidden relationship between the time series is automatically adjusted and learned through the neural network unit of the hidden layer and the correlation weight relationship between the units. This is because neural networks have the ability to dynamically adjust and learn from memory. A recurrent neural network consists of a hidden state and an output corresponding to the input sequence and is trained to predict the next output in the sequence. A recurrent neural network can learn a probability distribution over a sequence that maximizes that probability to output the most accurate next predicted value. The encoder-decoder structure is used to transform the input sequence through a series of functions of the model and output it. The length of the input sequence and the output sequence can be unequal, and the conditional distribution probability on the variable-length sequence can be learned through training.

(11) $$\begin{array}{c} b_{j}=\frac{1}{1+e^{-s}}=\frac{1}{1+e^{-\left(\sum_{i=1}^{n}w_{kj}x_{i}-\theta_{j}\right)}}. \end{array}$$

This formula indicates that the output function of each layer of neurons adopts the logsig function.

National economic accounting itself is a complex system engineering involving many fields and easily affected by various factors such as external environment and policy changes. Therefore, it is necessary to adopt an effective *FCM-BP* neural network model algorithm to strengthen the dynamic management and supervision of information. This model algorithm can continuously improve the level of national economic accounting and provide a better reference for the formulation of relevant national decisions.

## 4. Prediction Results from *FCM-BP* Model

**Figure 4:**
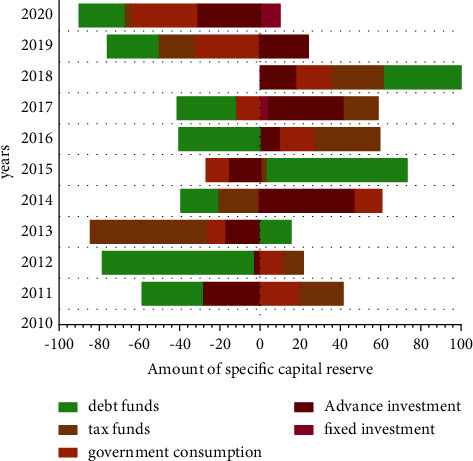
Reserves of various types of funds.

From Figure 4, we can see that the distributed data of average amount of tax for regions with different population are used to form the data acquisition terminal. To ensure that the effective data monitored must be complete, accurate, and transmitted to the data center in real-time situation, the stored data are uploaded for measuring instruments. The neural network mining tax prediction model uses an encoder-decoder sequence-to-sequence structure of recurrent neural networks, and the decoder part of the model is used to extract feature vectors from historical tax data and transmit them along the neural network link to model decoder section. The emergence of the Internet of Things makes modern society have the characteristics of the *FCM-BP* neural model.

The same is true of national accounts. First of all, the Internet of Things has made the national economic accounting into the “watchtower” of the *FCM-BP* neural model, which can realize real-time monitoring of economic activities. Second, the national economic accounts in the watchtower can learn under the supervision of the *FCM-BP* neural model and then continue to create, innovate, and improve. Third, the application of the Internet of Things in China's national economic accounting is actually equivalent to a small program of the *FCM-BP* neural model. The internal calculation algorithm can monitor the running state of the national economic accounting itself all the time. There is no doubt that the emergence of the Internet of Things has strengthened the important role of national accounts and promoted their self-learning and innovation capabilities. But conversely, if the national accounts let the Internet of Things go unchecked, it may create a series of problems that endanger information security and privacy protection.

(12) $$\begin{array}{c} {\hat{X}}^{\left(1\right)}\left(k+1\right)=\left[{\hat{X}}^{\left(0\right)}\left(0\right)-\frac{\hat{u}}{\hat{a}}\right]e^{-ak}+\frac{\hat{u}}{\hat{a}}. \end{array}$$

This is the predictive model algorithm obtained by solving differential equations.

**Figure 5:**
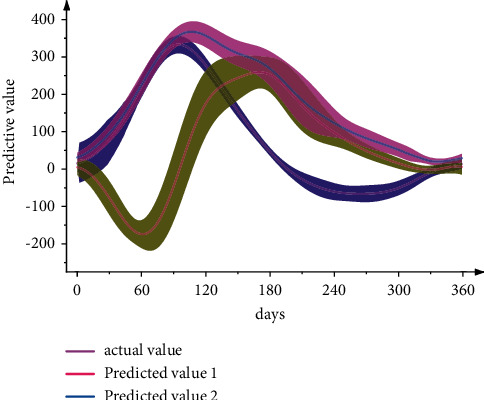
Model predicted values vs. actual values.

The prediction accuracy performance of the tax forecasting model will be evaluated based on the mean percentage error (*MAPE*) and the Pearson correlation coefficient (*R*). As shown in Figure 5, *MAPE* measures the size of the error by calculating the average of the relative error between the predicted value and the true value.

**Figure 6:**
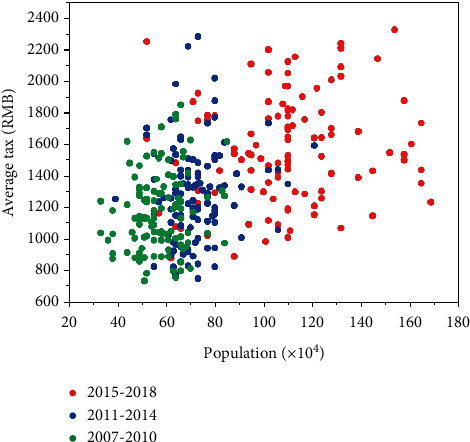
Average amount of tax for regions with different population bases.

The tax prediction model of *LSTM* (long short-term memory) neural network product without wavelet denoising on the tax data has a large deviation from the expected value on the seventh day. However, the predicted value of the product tax prediction model using wavelet noise reduction on the tax data does not have an extreme deviation from the expected mineral product tax value, the relative percentage error *MAPE* increases, and the linear correlation *R* decreases. It shows that the introduction of wavelet transform in the previous data for data denoising reduces the interference of the noise data in the original time series to the model prediction to a certain extent, and the prediction accuracy is improved by 7.12% compared with the model without noise reduction. Moreover, the correlation strength between the predicted value and the historical data is also improved compared with the model without data denoising. As shown in Figure 6, under *IoT* conditions, noise reduction records are the most fundamental information, while price information is additionally available through transaction systems. In other words, the Internet of Things era has naturally formed a separate pattern of price and quantity accounting, and the difficulty of quantity and price accounting will be greatly reduced.

**Figure 7:**
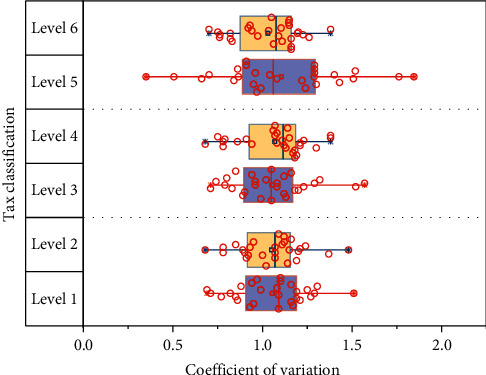
Calculation results of three levels of environmental monitoring.

The decoder part of the model predicts the taxation of mineral products in the future time period by decoding the feature vector.  Figure 7 shows the monitoring and calculation results of various levels of the national economic accounts. The historical tax data are a one-dimensional time series financial data, and the simple single-layer encoder-decoder sequence prediction model has limited ability to extract the hidden state and reconstruct the feature state vector of the one-dimensional time series. It shows the monitoring calculation results of each level of the calculation model. In addition, simply increasing the number of hidden nodes to expand the network capacity will lead to overfitting of the model, resulting in a large deviation in the prediction of future real values.

The national economic forecasting model based on the FCM-BP neural network without the encoder-decoder structure is constructed for the moving mode of economic forecasting. Move one time, and use the new time window sequence to predict the output at the next time, and shift back one time again, recursively until the entire output sequence is predicted. The *FCM-BP* neural network prediction model will accumulate the prediction error of the previous moment. Since the FCM-BP neural network prediction model will accumulate the prediction error of the previous moment, the error gradually accumulates as the window moves backward.

## 5. Evaluation of Models and Methods

**Figure 8:**
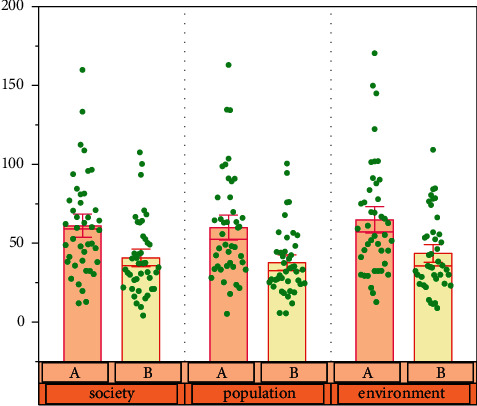
The impact of society, population, and environment on economic accounting.

The *FCM-BP* neural network evaluation method has the characteristics of high-speed self-learning, self-adaptation, fault tolerance, and flexibility. The information incompleteness evaluation system has greater advantages. Figure 6 shows the changes of two economic benefits over time. When the analysis object of internal control evaluation is fuzzy, incomplete, and uncertain, the sample data can be used for sufficient training and testing, and the evaluation results can be obtained through effective training. However, on the other hand, neural networks tend to converge slowly and require a lot of time for training.  Figure 8 shows the impact of social, demographic, and environmental factors on economic accounting. Due to the complexity of network structure and algorithm, it is difficult to understand and master, and there are high requirements for technical level. Moreover, when there are too many influencing factors and levels of internal control evaluation objects, with the increase of training times, the amount of calculation and storage capacity will also increase, and there may be over fitting, so it is impossible to obtain accurate evaluation results and predicted values.

This paper uses the constructed *FCM-BP* neural network to forecast the national economic accounting model to predict the tax revenue of the national economy. The input data are divided into two categories: those with wavelet noise reduction and those without noise reduction, to verify the wavelet noise reduction. Whether it has a positive role in improving the prediction accuracy of the model, and using the single-step sliding window *FCM-BP* prediction model and the *GM* (1, 1) model and the *ARIMA* model to do comparative experiments, to further verify the *FCM-BP* model in the national economic tax prediction. Ability to predict.

**Figure 9:**
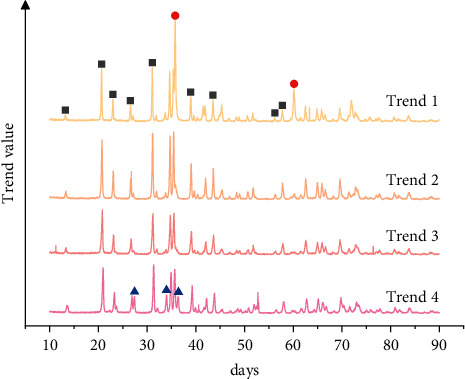
Economic forecast income trend chart.

As shown in Figure 9, the economic forecast trend chart of the four regions also provides us with a more comprehensive understanding of the complex issues in the development of various fields. The sample data used for the recurrent neural network tax prediction model are the national product tax data of a city from 2019 to 2021. The sample data are divided into training data for training, which is the tax data from 2019 to 2021. The prediction accuracy of the evaluation model is thus well verified.

**Figure 10:**
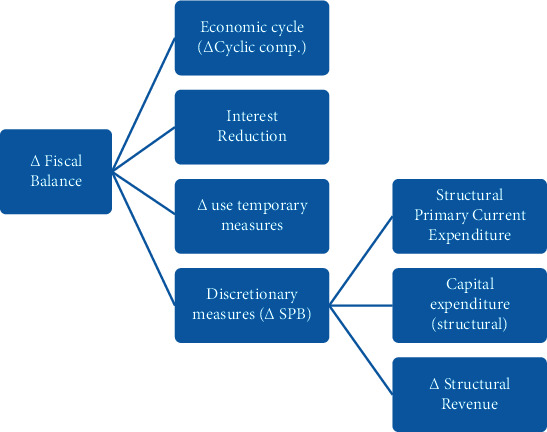
Decomposition of the budgetary balance.

Figure 10 gives the information about the decomposition of the budgetary balance. Budget balance is the balance between budgetary revenue and expenditure at various levels at the end of the year. Public finance is the direction and target of fiscal management and reform and the fundamental guarantee to realize the efficiency of fiscal fund allocation. The problem of excessive stock of current surplus funds reflects the weakening of budget execution of budget units, on the other hand, it reflects the rationalization of financial fund allocation to a certain extent. In the process of carrying out national accounts, it is often recorded by monetizing economic flows. In this case, much of the record work reflects mixed changes in price and quantity and does not accurately reflect their respective changes. As the quantity record is the most basic information, the application of Internet of Things technology can fully reflect the change of price information through the analysis of the transaction system. The model is trained separately by normalizing the data of the wavelet denoised data and the training data without denoising. The number of model training is 500 times, and the batch size of each grab for training is 32. The learning rate is dynamically adjusted using the Adam optimizer. The initial learning rate is set to 0.01. In order to prevent the model from overfitting, the dropout of the model is set to 0.2. The loss function uses the mean square logarithmic loss (*MSLE*) function. Therefore, in the application of the Internet of Things technology, a discrete pattern is formed between the quantity and the price through the *FCM-BP* neural network, which can greatly reduce the difficulty of quantity accounting and price accounting. As shown in Figure 10, especially in this case, the price of the commodity itself and the growth data of the material quantity can be provided separately through the Internet of Things technology, which can greatly promote the accuracy of policy analysis and market analysis so as to ensure the stable development of China's economy to the greatest extent. IoT and economic settlement depth fusion make financial relying on Internet technology, improving service experience, to reduce the operating costs, cash flow, information flow, and physical flow of third-rate unity, thereby change the financial credit system, control the financial risk, profoundly change the banking, securities, insurance, leasing, investment, and many other financial sector of the original model and bring about new financial revolution.

Substitute the obtained data into the neural network prediction model established by the national economic income. The prediction results of the neural network prediction model shows the comparison between the actual value and the predicted value of tax revenue under the neural network model. It can be seen that the predicted value has a good fit with the original value, so the *FCM-BP* neural network model is used to predict the national tax revenue, and the prediction accuracy is high. The identification of Internet of Things assets is a necessary and key link in Internet of Things security. If the exposed Internet of Things assets can be summarized and analyzed, it is of great significance to discover the security problems of Internet of Things devices. By means of clustering calculation, experience can be used to improve the performance of the Internet of Things system itself.

## 6. Conclusion

The national economic forecasting model based on *FCM-BP* neural network proposed in this paper. Although the average absolute percentage error of the prediction results in the medium and long term is low, there are large errors at individual time nodes. For general unidirectional time-series data, FCM-BP neural network model can only extract limited hidden information from the data. Although the FCM-BP algorithm is not as perfect as the traditional optimization algorithm in theory, such methods do not require a clear analytic formula for the objective function and have strong adaptability to the uncertainty of data in calculation, so they are suitable for solving Big Data optimization problems. The *FCM-BP* neural network combined with the Internet of Things technology has a broad and far-reaching impact on the accounting rules, accounting framework, and accounting products of the national economic accounting. The research in this paper is also based on the existing development trend of the Internet of Things and the analysis framework of national economic accounts. Using BP neural network, the forecasting method of national economy is proposed and verified by actual data. The *FCM-BP* neural network model of the article has high precision for future economic forecast.

## Data Availability

The data used to support the findings of this study are available from the corresponding author upon request.
